# Supplementary material for: Deuterium-Depleted Water in Cancer Therapy: A Systematic Review of Clinical and Experimental Trials
Source: Nutrients. 2024 May 6;16(9):1397. doi: 10.3390/nu16091397 (PMC11085166; doi:10.3390/nu16091397)
Supplement: Supplementary file 1 [file nutrients-16-01397-s001.zip › nutrients-2969371-supplementary.pdf]

WEBAPPENDIX FOR: “Deuterium-depleted water in the Cancer Therapy: A Systematic Review of Clinical and Experimental trials.” Detailed search strategy

PubMed publisher

(("deuterium"[MeSH Terms] OR "deuterium"[All Fields] OR "deuteriums"[All Fields]) AND ("deplete"[All Fields] OR "depleted"[All Fields] OR "depletes"[All Fields] OR "depleting"[All Fields] OR "depletion"[All Fields] OR "depletions"[All Fields]) AND ("water"[MeSH Terms] OR "water"[All Fields] OR "watering"[All Fields] OR "water s"[All Fields] OR "watered"[All Fields] OR "waterer"[All Fields] OR "waterers"[All Fields] OR "waterings"[All Fields] OR "waters"[All Fields]))

Web-of-science

((TS=(deuterium deplet*)) AND TS=(water))

Cochrane

(deuterium deplet*)
